# Synergistic interaction of a consortium of the brown-rot fungus *Fomitopsis pinicola* and the bacterium *Ralstonia pickettii* for DDT biodegradation

**DOI:** 10.1016/j.heliyon.2020.e04027

**Published:** 2020-06-07

**Authors:** Adi Setyo Purnomo, Atmira Sariwati, Ichiro Kamei

**Affiliations:** a Department of Chemistry, Faculty of Science and Data Analytics, Institut Teknologi Sepuluh Nopember (ITS), Kampus ITS Sukolilo, Surabaya, 60111, Indonesia; b Department of Tiongkok Traditional Medicine, Faculty of Health Science, Institut Ilmu Kesehatan Bhakti Wiyata Kediri, Jalan KH Wahid Hasyim 65, Kediri, 64114, Indonesia; c Department of Forest and Environmental Sciences, Faculty of Agriculture, University of Miyazaki, 1-1 Gakuen-kibanadai-nishi, Miyazaki, 889-2192, Japan

**Keywords:** Biotechnology, Microbiology, Pesticide, Environmental chemistry, Environmental pollution, Microorganism, Microbial biotechnology, Biodegradation, DDT, *Fomitopsis pinicola*, *Ralstonia pickettii*, Synergistic, Microbial consortium

## Abstract

1,1,1-Trichloro-2,2-bis (4-chlorophenyl) ethane (DDT) is a toxic and recalcitrant pesticide that has been greatly used to eradicate malaria mosquitos since the 1940s. However, the US Environmental Protection Agency banned and classified DDT as priority pollutants due to its negative impact on wildlife and human health. Considering its negative effects, it is necessary to develop effective methods of DDT degradation. A synergistic interaction of a consortium consisting of the brown-rot fungus *Fomitopsis pinicola* and the bacterium *Ralstonia pickettii* was adopted to degrade DDT. For the microbial consortia, *F. pinicola* was mixed with *R. pickettii* at 1, 3, 5, 7 and 10 ml (1 ml ≈ 1.44 × 10^13^ CFU) in a potato dextrose broth (PDB) medium to degrade DDT throughout the seven days incubation period. The degradation of DDT by only the fungus *F. pinicola* was roughly 42%, while by only *R. pickettii* was 31%. The addition of 3 ml of *R. pickettii* into *F. pinicola* culture presented appropriate optimization for efficient DDT degradation at roughly 61%. The DDT transformation pathway by co-inoculation of *F. pinicola* and *R. pickettii* showed that DDT was converted to 1,1-dichloro-2,2-bis(4-chlorophenyl) ethane (DDD), further transformed to 1,1-dichloro-2,2-bis(4-chlorophenyl) ethylene (DDE), and then ultimately transformed to 1-chloro-2,2-bis(4-chlorophenyl) ethylene (DDMU). These metabolites are less toxic than DDT. This research showed that *R. picketti* synergistically interacts with *F. pinicola* by enhancing DDT degradation.

## 1 Introduction

Fungi and bacteria are the main groups of organisms with regard to both biomass and metabolic processes, and the majority of fungi and bacteria are degradation microbes, several substrates are noticeable without difficulty to microbes (De Boer et al., 2005; Meidute et al., 2008). Fungi and bacteria colonies degrade cell wall components such as lignin. Lignin is structurally similar to many organic pollutants, and so many fungi and bacteria have great potential for use in the biotransformation of organic pollutants (Kamei et al., 2012). Fungus thrives according to climatic requirements such as temperature, water content (moisture conditions), pH, and abundance of nutrients that can lead to rapid proliferation in the soil matrix (Ramirez et al., 2010). In general, fungi thrive better than bacteria because they have extracellular enzymes that can be adapted to trounce high molecular weight pollutants in the soil, releasing extracellular enzymes that can diffuse in the soil (Wang et al., 2012; Ramadhania et al., 2018; Nawfa et al., 2019). Besides, fungi have the ability to synthesize general enzymes for the degradation of complex mixtures of toxic substances (Husaini et al., 2008; Ma et al., 2015; Hidayati et al., 2017; Laili et al., 2018). Most fungi are microbes capable of being tolerant with the concentration of toxicants more than bacteria (Gianfreda and Rao, 2004). Conversely, bacteria could have an elevated affinity for organic substrates (Romani et al., 2006), because they utilize carbon, leading to the degradation of hydrocarbons (Hamsavathani et al., 2015). Bacteria increase cellular uptake of pollutant compounds, manipulate substrate by ring fission and ring cleavage, convers cleaved product into intermediate metabolites, and further utilize intermediate metabolites by using enzymes to initiate significant mechanism for degradation (Thakur, 2007).

Fungi and bacteria mostly partake in consubstantial ecology (Warmink and Van Elsa, 2009) and interact with each other (Ellegard-Jansen et al., 2014), as this interaction is important to achieve synergism and competition within varying microorganisms (Lade et al., 2012). Recently, varying consortia advances have been reported, with strengthened potential for degradation due to the combined and inductive effect of various enzymes. Several studies have been conducted on the capability of co-culture of fungi and bacteria to increase the pollutants degradation such as benzopyrene (Ramirez et al., 2010), high molecular aromatic compounds (Husaini et al., 2008; Ma et al., 2015), pesticides (Ellegard-Jansen et al., 2014), and polyaromatic hydrocarbons (Boonchan et al., 2006).

DDT (1,1,1-trichloro-2,2-bis(4-chlorophenyl) ethane) is a persistent compound with a half-life of 15 years, in consequence framing it steady in the environment (Wang et al., 2010). The DDT had been prohibited in certain nations since 1970 (Bidlan and Mannonmani, 2002), as DDT residue was found in ground and surface water used for potable water supply (Hai et al., 2012) and in most soil (Wang et al., 2010). DDT residues are oleophilic in nature, contributing to the accumulation in adipose tissues of the feeding organisms throughout the food chain (Bidlan and Mannonmani, 2002), which have been linked to adverse non-communicable diseases such as cancer and numerous reproductive defects (Ahmad et al., 2010; Fang et al., 2010). To overcome these associated disorders, eco-friendly techniques of bioremediation have been suggested.

The brown-rot fungus (BRF), *Fomitopsis pinicola*, has been shown to have fabulous leverage on the DDT degradation (Purnomo et al., 2008, 2010c, 2011b; Sariwati et al., 2017; Sariwati and Purnomo, 2018), through oxidoreductase (Oprica et al., 2008) and laccase (Park and Park, 2014). Similarly, *Ralstonia pickettii* possesses significant bioremediation potential, through its demonstrated ability to break down xenobiotic pollutants that contain aromatic hydrocarbons (e.g. toluene, trichloroethylene, and chlorobenzene) (Ryan et al., 2007; Zhang et al., 2011). Notably, *R. pickettii* produces biosurfactants belonging to the major class of glycolipids-rhamnolipids (Plaza et al., 2007; Sukandar et al., 2016), aggregating surfactant molecules to ease the accessibility of the organic compounds to the microorganisms (David et al., 1991; Fatmawati et al., 2015).

The combination of fungi and bacteria has no absolute limitation, as regards their ability to degrade xenobiotic pollutants. Therefore, the consortium of *F. pinicola* and *R. pickettii* may produce synergistic effect to enhance DDT degradation. In this research, the capability of the consortium of *F. pinicola* and *R. pickettii* for degrading DDT was observed to recognize the metabolic products and conversion routes of DDT.

## 2 Materials and methods

### 2.1 Chemicals

DDE, DDT, DDMU, DDD, and pyrene were purchased from Tokyo Chemical Industry Co., while methanol, dimethylsulfoxide (DMSO), and anhydrous sodium sulfate were purchased from Merck Millipore (Darmstadt, Germany). Lastly, acetone and *n*-hexane were purchased from Anhui Fulltime Specialized Solvent & Reagent Co. Ltd (Anhui, China).

### 2.2 Fungus culture condition

The *F. pinicola* NBRC 8705 (NITE Biological Resources Center (NBRC), Chiba, Japan) was cultivated as stock culture in a 9-cm diameter plates containing potato dextrose agar (PDA; Merck Darmstadt, Germany) at 30 °C. A 1-cm diameter of mycelia from stock culture was inoculated into a 10 ml potato dextrose broth (PDB; Merck Darmstadt, Germany) medium in a 100-ml Erlenmeyer flask, and then was pre-incubated at 30 °C for 7 days (Rizqi and Purnomo, 2017; Purnomo et al., 2017a; Boelan and Purnomo, 2019).

### 2.3 Bacterium culture condition

The stock suspension of *R. pickettii* NBRC 102503 was cultivated at 37 °C in a nutrient agar (NA, Merck, Darmstadt, Germany). The formed colony was inoculated into a 100-ml Erlenmeyer flask containing 100 ml of nutrient broth (NB, Merck, Darmstadt, Germany) medium. The suspension was pre-incubated at 37 °C on a shaker (WINA Instrument, type 102B, Indonesia) at 180 rpm for 30 h (Wahyuni et al., 2016, 2017).

### 2.4 DDT degradation by *F. pinicola*

After pre-incubation for 7 days, 10 ml of PDB medium was added into inoculated *F. pinicola* cultures (final volume 20 ml), and 50 μL of 5 mM DDT in DMSO was added to each *F. pinicola*-inoculated flask. The cultures were further incubated for 7 days at 30 °C, while the control cultures were autoclaved (121 °C, 15 min) after pre-incubation (Setyo et al., 2018; Purnomo et al., 2019b).

### 2.5 DDT degradation by *R. pickettii*

After pre-incubation for 30 h, *R. pickettii* cultures were inoculated into the PDB medium at 1, 3, 5, 7 and 10 ml (1 ml ≈ 1.44 × 10^13^ CFU, ultimate volume = 20 ml). Fifty microliters (50 μl) of 5 mM DDT in DMSO was added in every bacteria-inoculated flask. The cultures were incubated for 7 days at 30 °C, while the control cultures were annihilated by autoclave (121 °C, 15 min) after pre-incubation (Purnomo, 2017).

### 2.6 DDT degradation by co-cultures of *F. pinicola* and *R. pickettii*

After pre-incubation of *F. pinicola* and *R. pickettii* for 7 days and 30 h respectively, *R. pickettii* cultures were inoculated separately at 1, 3, 5, 7 and 10 ml (1 ml ≈ 1.44 × 10^13^ CFU) into *F. pinicola* cultures in the PDB medium (reaching an ultimate volume of 20 ml). Fifty microliters (50 μl) of DDT 5 mM in DMSO was added in each inoculated flask. The suspension was incubated for 7 days at 30 °C, while the control cultures were annihilated by autoclave (121 °C, 15 min) in after pre-incubation. The best combination of co-cultures for DDT degradation was recommended for further additional experiments (Purnomo, 2017; Purnomo et al., 2019a). For the establishment of the degradation pathway, DDT metabolites (DDE, DDD, and DDMU) were used as substrates. The synergistic relationship of co-cultures was expressed with Ratio Optimization (RO), which is a calculation of the degradation rate by co-cultures per total degradation rate by fungi and bacteria.

### 2.7 Analytical method

After incubation, 50 μl of pyrene in 5 mM DMSO was added as an internal standard, homogenized with 20 ml of methanol, washed with 5 ml of acetone, and then centrifuged at 3000 rpm for 10 min. Following centrifugation, the supernatant was filtered using Whatman filter paper 41 (GE Healthcare Life Science, UK), separating it from the biomass. The filtrates were evaporated at 64 °C to remove methanol and acetone, then extracted using 200 ml *n*-hexane, and then the organic fraction was collected and dried in anhydrous sodium sulfate. The extracts were evaporated at 68 °C using an evaporator. The concentrate was diluted with methanol, after which it was analyzed by high-performance liquid chromatography (HPLC; Shimadzu, Japan) to measure the total DDT and its metabolites. The HPLC operation incorporated a LC- 20AT pump (Shimadzu, Japan) with a SPD-M20A diode array detector (Shimadzu, Japan), matched with an inertsil ODS-3 column (150 mm) with an inside radius of 4.6 mm (GL Science, Japan). The samples were eluted with 82% methanol in a 0.1% trifluoroacetic acid aqueous solution, at an inflow rate of 1 ml min^-^^1^. DDT and its metabolites were detected at the base of the retention time, with optimal absorption at particular wavelengths similar to the standard. For quantitative examination, the peak area of DDT and its metabolites were compared with the peak area of pyrene (Purnomo et al., 2010c). To identify metabolites that could not be detected by HPLC, the samples were diluted with *n*-hexane and then analyzed by gas chromatography/mass spectrometry (GC/MS; HP, USA). GC/MS was accomplished on an HP 6890 GC system (HP, USA) linked to an HP 5973 mass-selective sensor (HP, USA), with a 30-m fused DB-5MS column (J&W Scientific, CA, USA). The injector temperature was designed at 250 °C, as the injection was splinted close to 1 μl. The oven temperature was set at 80 °C for 3 min, and then increased linearly to 300 °C at 20 °C min^-^^1^, hold for 5 min (Purnomo et al., 2010a, 2010b, 2011b).

### 2.8 Synergism activity of the whole culture

*R. pickettii* was pre-incubated in the nutrient broth (NB) at 37 °C for 30 h (concentration 10^13^ CFU/ml). The bacterial inoculum was put on one side of the petri dish containing of the PDA, after which the 1-cm diameter agar plug containing of the fungal mycelium *F. pinicola* was placed at the midpoint of the plate, and then incubated for 3 days at 30 °C. The radius of the fungal colony within the bacterial colony was determined daily (Joshi et al., 2008; Kamei, 2017).

### 2.9 Statistical data analysis

All data values were the average of triplicate determinations expressed with the standard deviation (SD). The normality and paired sample t-tests were carried out to analyze the significant differences between the various treatments using SPSS 22 for Windows (SPSS Inc., USA), with the significance level estimated at 5% (p < 0.05) (Purnomo et al., 2013, 2014).

## 3 Results

This study showed significant synergistic interaction between the consortium of *F. pinicola* and *R. pickettii* during the biodegradation of DDT, compared to the individual strains. Biodegradation of DDT by different volumes of *R. picketti* for the 7 days’ incubation term in PDB medium is shown in Table 1. About 31% of DDT was degraded (the greatest DDT biodegradation) by 7 ml (1.44 × 10^13^ CFU/ml) of *R. pickettii*, which was significantly lower compared to the degradation of roughly 42% of DDT achieved by pure fungal *F. pinicola* strain (P < 0.05; supplementary 1). This result indicates that, individually, both the bacterium and fungus were not very efficient in DDT degradation.

**Table 1:** Degradation of DDT by individual cultures and co-cultures of *F. pinicola* and *R. pickettii* in PDB medium during the 7-day incubation.

Amount of *R. pickettii* (ml)	Degradation of DDT (%)		Ratio Optimization
	*R. pickettii*	Co-cultures	
(*F. pinicola only*)	41.72 ± 1.54^a^		
1	8.62 ± 0.65^bA^	54.47 ± 0.24^gB^	1.08
3	12.08 ± 0.88^cA^	61.44 ± 2.2^hB^	1.14
5	15.64 ± 0.29^dA^	56.21 ± 0.84^iB^	0.98
7	30.87 ± 2.29^eA^	72.85 ± 1.24^jB^	1.00
10	19.84 ± 0.60^fA^	65.66 ± 0.50^kB^	1.06

Analyses were conducted by HPLC. Data are mean ± standard deviation (n = 3). A 1 ml of bacteria ≈1.44 × 10^13^ bacteria cell/ml culture. Data followed by the same minor letter on each column or by the same capital letter on each row are not significantly different (*P < 0.05*).

**Figure 1:**
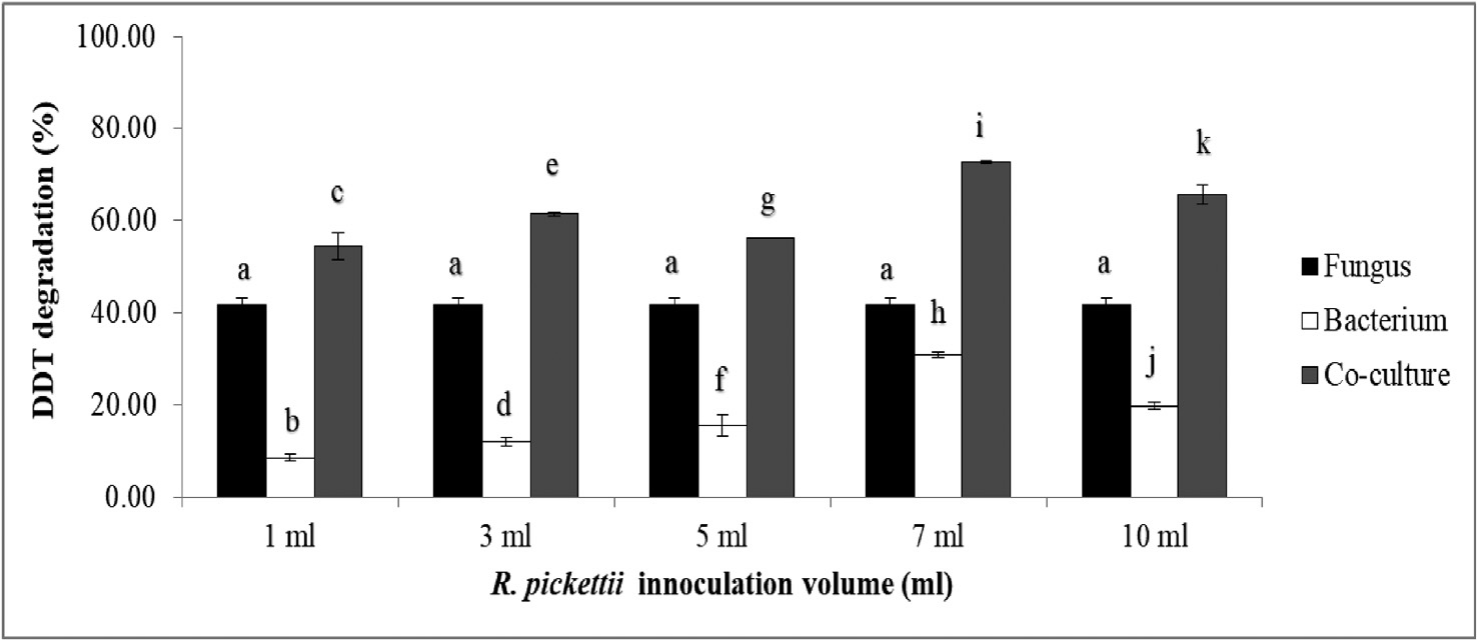
Degradation rate of DDT during the 7-day incubation. A 1 ml of *R. pickettii* inoculation volume equal to 1.44 × 10^13^ CFU. Data points are means and standard deviations (n = 3). Data followed by the same minor letter on each bar is not significantly different (*P < 0.05*).

The DDT biodegradation by the co-culture of *F. pinicola* and *R. pickettii* was observed and is shown in Table 1. About 54%, 61%, 56%, 73%, and 66% of DDT were degraded by the consortia of *F. pinicola* and *R. pickettii* at 1, 3, 5, 7 and 10 ml (1.44 × 10^13^ CFU/ml) respectively. The highest amount of DDT degraded was roughly 73% for the 7 ml (1.44 × 10^13^ CFU/ml) input of *R. pickettii*. On the whole, the DDT biodegradation by the fungal-bacterial consortium was the most significant efficient approach (p < 0.05, supplementary 1). Figure 1 shows that DDT degradation by *F. pinicola* significantly improved when combined with 1, 3, 5, 7 and 10 ml (1.44 × 10^13^ CFU/ml) of *R. pickettii* (P < 0.05, supplementary 1). Hence, the combined *F. pinicola* with 7 ml (1.44 × 10^13^ CFU/ml) of *R. pickettii* formed the most effective degradation.

The synergistic relationship between *F. pinicola* and *R. pickettii* during the DDT degradation was expressed with Ratio Optimization (RO) (Table 1). The mixed *F. pinicola* with 3 ml (1.44 × 10^13^ CFU/ml) of *R. picketti* showed the highest RO of 1.14, thus additional experimentation to factor in the extra time for *R. pickettii* addition was recommended to detect metabolic products and the conversion route.

**Figure 2:**
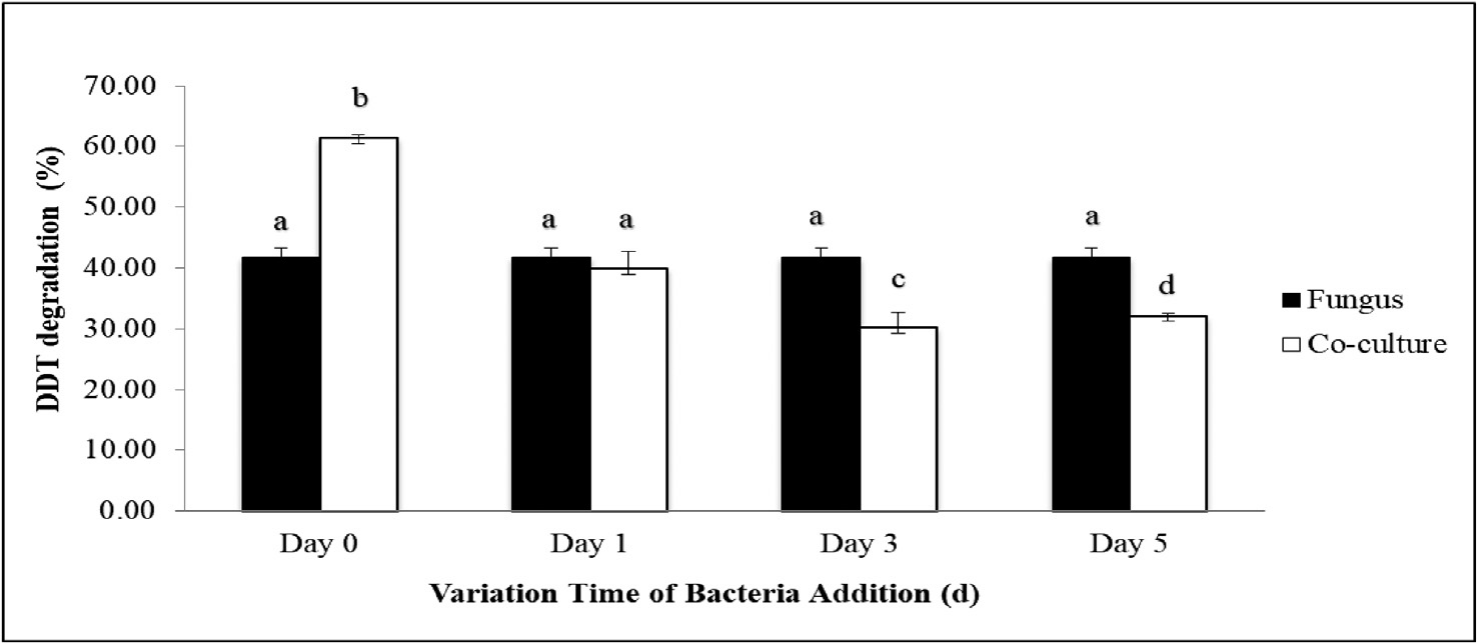
The addition time of *R. pickettii* on DDT degradation. Data points are means and standard deviations (n = 3). The same minor letter on each bar indicates no significant difference (*P < 0.05*).

The outcomes of the alteration in extra-time of *R. pickettii* addition into the fungus culture at the 0^th^, 1^st^, 3^rd^, and 5^th^ day are shown in Figure 2. Notably, the introduction of *R. pickettii* into the fungus suspension at concurrent (0 d) was significantly the most efficacious time for topmost DDT degradation (61%, P < 0.05, Supplementary 2). In contrast, the introduction of the bacteria on the 3^rd^ day into the *F. pinicola* suspension resulted in minimum DDT degradation by a significant 30% (P < 0.05, Supplementary 2).

**Figure 3:**
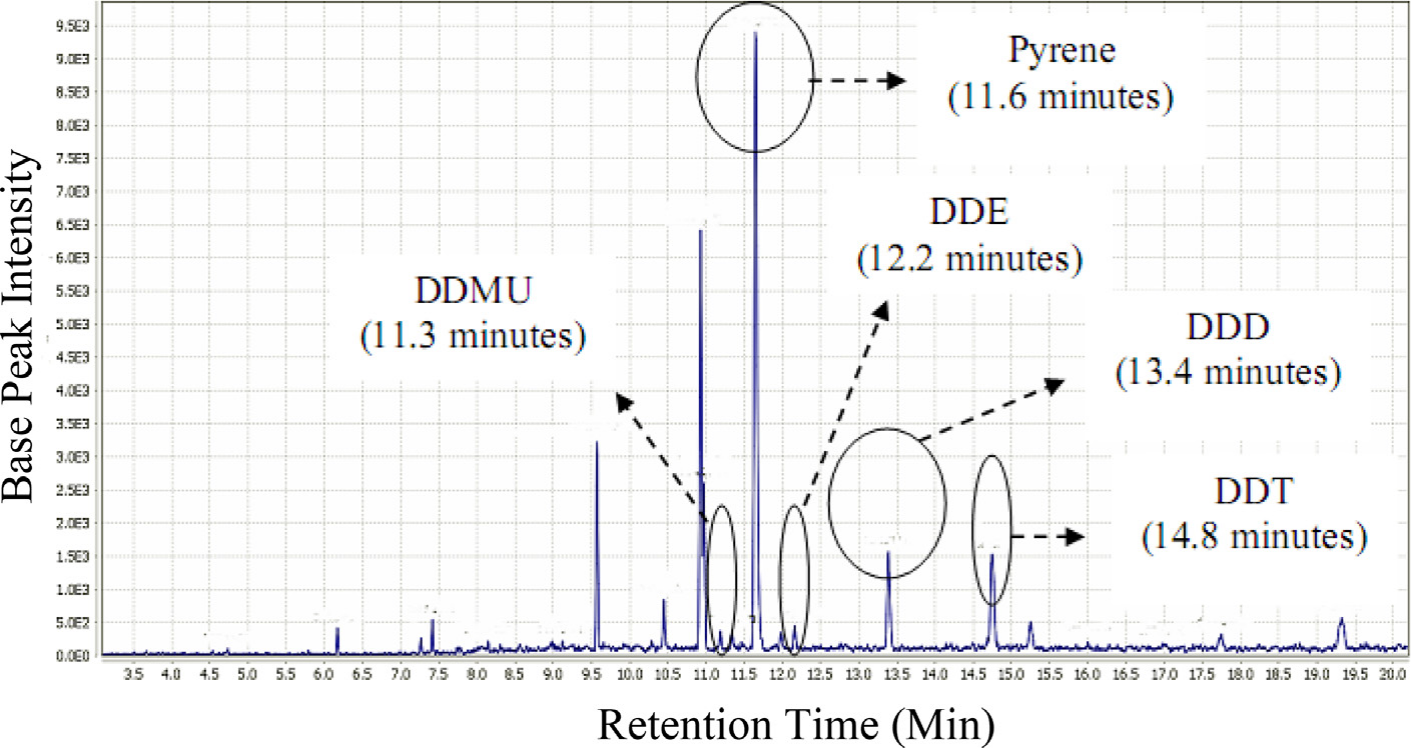
Chromatogram of DDT degradation by co-cultures of *F. pinicola* and *R. pickettii* during the 7-day incubation.

The ability of the consortium of *F. pinicola* and *R. pickettii* to transform DDT to DDD, DDE, and DDMU is shown in Figure 3, whilst the recognized recovered metabolic products are presented in Table 2. The metabolic product DDD was encountered as the primary product, constituting roughly 51% of the total products (P < 0.05), while DDE and DDMU were significant slight metabolites generated. DDD was degraded by the consortium by about 50% (P < 0.05), leaving DDE and DDMU as the detected metabolites. Furthermore, the consortium also degraded DDE by about 55% (P < 0.05), resulting in DDD and DDMU as the only distinguished metabolic products, although the concentration of DDMU was, however, higher than DDD, suggesting that DDE was probably transformed to DDMU.

**Table 2:** Recovered DDT and its metabolic products by co-cultures of *F. pinicola* and 3 ml *R. pickettii*.

Substrates	Degradation (%)	Recovered metabolic products (%)			
		DDD	DDE	DDMU	Total
DDT	61.44 ± 2.21^a^	51.18 ± 1.10^b^	0.52 ± 0.60^c^	1.30 ± 1.01^c^	53.00 ± 2.71
DDD	49.61 ± 4.33^a^	-	0.89 ± 0.39^b^	0.14 ± 0.09^c^	1.03 ± 0.48
DDE	54.65 ± 5.68^a^	1.47 ± 0.60^b^	-	5.21 ± 3.89^c^	6.68 ± 4.49

Analyses were conducted by HPLC. Data are mean ± standard deviation (n = 3). Data followed by the same minor letter on each row are not significantly different (*P < 0.05*).

## 4 Discussion

Environmental pollutants are caused by releases from industries, like the chemical, pharmaceutical and agricultural (enhancement products like pesticides, especially DDT) industries. Biodegradation of DDT by microorganisms is one of the substantial approaches, to remove the environmental harmful compounds. The ability of fungi and bacteria to metabolize DDT has been vastly acknowledged as a competent means to end its poisonous and dangerous discharge.

Notably, *F. pinicola* strain degraded DDT by approximately 42%, with DDD and DDE distinguished as its metabolite products, during the 7-day incubation in the PDB medium. This result contrasts with that of a previous study (Purnomo et al., 2008), where *F. pinicola* degraded DDT by approximately 84%, after a cultivation term of 28 days in the PDB medium, generating DDD as its metabolic product. It was suggested that DDT was converted to DDD and DDE at the initial stage of degradation, while DDE was further transformed to DDD as the end product, although the period of incubation was longer. Furthermore, *R. pickettii* showed with increasing concentration of *R. pickettii* (Table 1), higher degradation of DDT was observed. The most effective degradation was achieved by *R. pickettii* at a concentration of 7 ml (1.44 × 10^13^ CFU/ml), which DDT was degraded by 31%. However, in excess concentration of *R. pickettii* (10 ml), DDT degradation was lower (20%), as it promotes its own survival at this concentration more than it degrades DDT. Bacteria produce some secondary metabolites when cultured at the stationary phase, which is deleterious to their independent survival. DDE was encountered as a metabolite of DDT degradation by *R. pickettii.*

Results from this study show that individual degradation of DDT by bacteria and fungi was low, as the detected metabolic products (DDD and DDE) were still recalcitrant toxic compounds. This results are similar to those of a study on DDT degradation by 4-chlorobiphenyl-degrading Gram-negative bacterium, in which DDT was mainly converted to DDD prior to dehydrogenation to DDE, which was followed by de-chlorination to DDMU under aerobic conditions (Masse et al., 1989). Similarly, the action of the ectomycorrhizal fungi *Xerocomus chrysenteron* on DDT resulted in DDT conversion to DDE (Huang and Wang, 2013).

As attempts to improve the degradation of DDT into less hazardous metabolites, the synergistic method of degradation by fungal-bacterial interaction is considered a feasible tool for the effective degradation of xenobiotic pollutants (Lade et al., 2012). The consortia consisting of both bacteria and fungi, their respective biomass accumulation, and their relative enzymes all work synergistically. This synergism is more efficient because of its focus on metabolic processes which possess the ability to breakdown pollutant molecules and appropriate transitional degradation metabolites for mineralization (Lade et al., 2012; Keck et al., 2002). The DDT degradation rates by each of the fungus *F. pinicola*, and bacterium *R. pickettii*, compared to the rates of their mixed cultures, are presented in Figure 1. The DDT degradation by the co-cultures was the highest, compared to the degradation by the fungi and bacteria separately. However, varying the amount of *R. pickettii* added into the *F. pinicola* culture affected the efficiency of DDT degradation (Figure 1), as 7 ml (1.44 × 10^13^ CFU/ml) of *R. pickettii* was found to be the most effective, degrading DDT by roughly 73% (Table 1). In excess concentration of *R. pickettii* (10 ml) there was a resultant decrease in DDT degradation due to the promotion of bacteria survivability, and at this concentration DDT was degraded by roughly 66% (Table 1).

Mixed cultures have been inspected to see if they are preferable to singular isolates for DDT degradation, as *R. pickettii* produces biosurfactant, belonging to the major class of glycolipids-rhamnolipids (Plaza et al., 2007), which increases the solubility of DDT (Aislable et al., 1997) and further results in easy uptake of DDT by *F. pinicola*. In previous report, the adjunction of 10 mL R*. pickettii* heighten DDT degradation by the brown-rot fungus *Daedalea* *dickinsii* (Setyo et al., 2018), and also a consortium of *R. pickettii* with *Pleurotus eryngii* likewise was found to enhance DDT degradation (Purnomo et al., 2019a). Several studies have also shown fungus-bacterium co-culture degradation of pyrene, phenanthrene, and fluoranthene by 67%, compared to degradation by 39% by cultures of fungal and by 56% by cultures of bacterial, with an extended incubation period of 28 days (Wang et al., 2012). Replacement of some pesticides by a co-culture of bacteria from sludge and the white-rot fungus *Coriolus versicolor* resulted in an immense drop in the concentration of aldicarb, atrazine, and alachlor (Hai et al., 2012). Fungal and bacterial consortium could also degrade endosulfan in an aqueous medium and in the soil (Abraham and Silambrasan, 2014). In an appurtenant research on the synergistic effect of a co-culture of the white-rot fungus, *Pleurotus ostreatus*, and the biosurfactant-intercourse bacteria, *Bacillus subtilis* and *Pseudomonas aeruginosa*, on DDT biodegradation (Purnomo et al., 2017b), it was found than an input of 3 ml of *P. aeruginosa* into *P. ostreatus* culture resulted in DDT degradation by 86% within a 7-day incubation period. Besides, the addition of 10 ml of *B. subtilis* inside white-rot fungus *Ganoderma lingzhi* was denounced heighten DDT degradation by about 82.30% throughout a 7-day incubation term (Grizca and Setyo, 2018). The ability of the consortium of the brown-rot fungus, *F. pinicola*, and *P. aeruginosa* to biodegrade DDT has also been reported, with the addition of 10 ml of *P. aeruginosa* to the fungal culture resulted in elevated biodegradation of DDT by 68%, within a 7-day incubation term (Sariwati and Purnomo, 2018). In another study, the addition of 10 ml of *B. subtilis* to *F. pinicola* culture resulted in a highly efficient DDT degradation by 86% within 7-day of incubation (Sariwati et al., 2017).

The effectiveness of *F. pinicola* and *R. pickettii* consortia in the degradation of DDT was regulated by Ratio Optimization (RO). RO pointed out the improvement in cooperative mechanisms between *F. pinicola* and *R. pickettii*, and the effect of individual fungal and bacterial strains on DDT degradation. Overall, the addition of varying concentrations of *R. pickettii* was denoted by RO > 1 (Table 1), except at a concentration of 5 ml, as the addition of 3 ml achieved the highest RO. This indicates that the input of 3 ml of *R. pickettii* into *F. pinicola* culture provided the optimum synergistic relationship, degrading the DDT by roughly 61%.

The synergistic interactions between fungi and bacteria also influence their combined degradative enzyme activities (Mikeskova et al., 2012). *F. pinicola* produces degradative extracellular enzymes through oxidoreductase, superoxide dismutase and catalase activities (Oprica et al., 2008; Fritsche and Hofrichter, 2005). Oxidoreductase has degradation capacity to convert insoluble compounds to incompletely oxidized products, which can then be effortlessly taken up by cells through enzymes that increase polarity and water solubility, further oxidizing poisonous dissolved products into insoluble cell structures (Gianfreda and Rao, 2004). Moreover, *F. pinicola* produces laccase (Park and Park, 2014), which utilizes molecular oxygen as an electron receiver to co-substrate and oxidize different aromatic and non-aromatic compounds through a radically mobilized response process (Dashtban et al., 2010; Singh et al., 2015), as well as produce P450 monooxygenase (Floudas et al., 2012). *F. pinicola* also produces peroxidase, which are oxidoreductases that catalyze the reduction of peroxides, such as hydrogen peroxide (H_2_O_2_) (Floudas et al., 2012; Bansal and Kanwar, 2013), and the oxidation of aromatic and halogenated phenolic compounds (Karigar and Rao, 2011).

*R. pickettii* produces monooxygenase renowned for bioremediation and its biocatalyst action in green chemistry (Fishman et al., 2005). Although *R. pickettii* produces lipase enzyme (Hemachander and Puvanakrishnan, 2000), the lipase is adsorbed on to the oil-water surface (hydrophobic property) despite the presence of water (Karigar and Rao, 2011). *R. pickettii* produces depolymerase (Hiraishi and Taguchi, 2009), which is one of the primary categories of oxidoreductase (Rao et al., 2010). In degradation by co-cultures of *R. pickettii*, fungal extracellular enzymes are detached to enable the penetration of molecules that are overly large to traverse beyond the bacterial cellular wall, attaining a partial oxidative degradation. The fungal metabolites are then degraded by bacteria to smaller molecules, by intracellular enzymes (Hammel, 1995; Lease, 2006). Furthermore, the action of *F. pinicola* and *R. pickettii* may be due to their cooperative catabolism, as *F. pinicola* transforms DDT into products that are used by *R. pickettii*.

**Figure 4:**
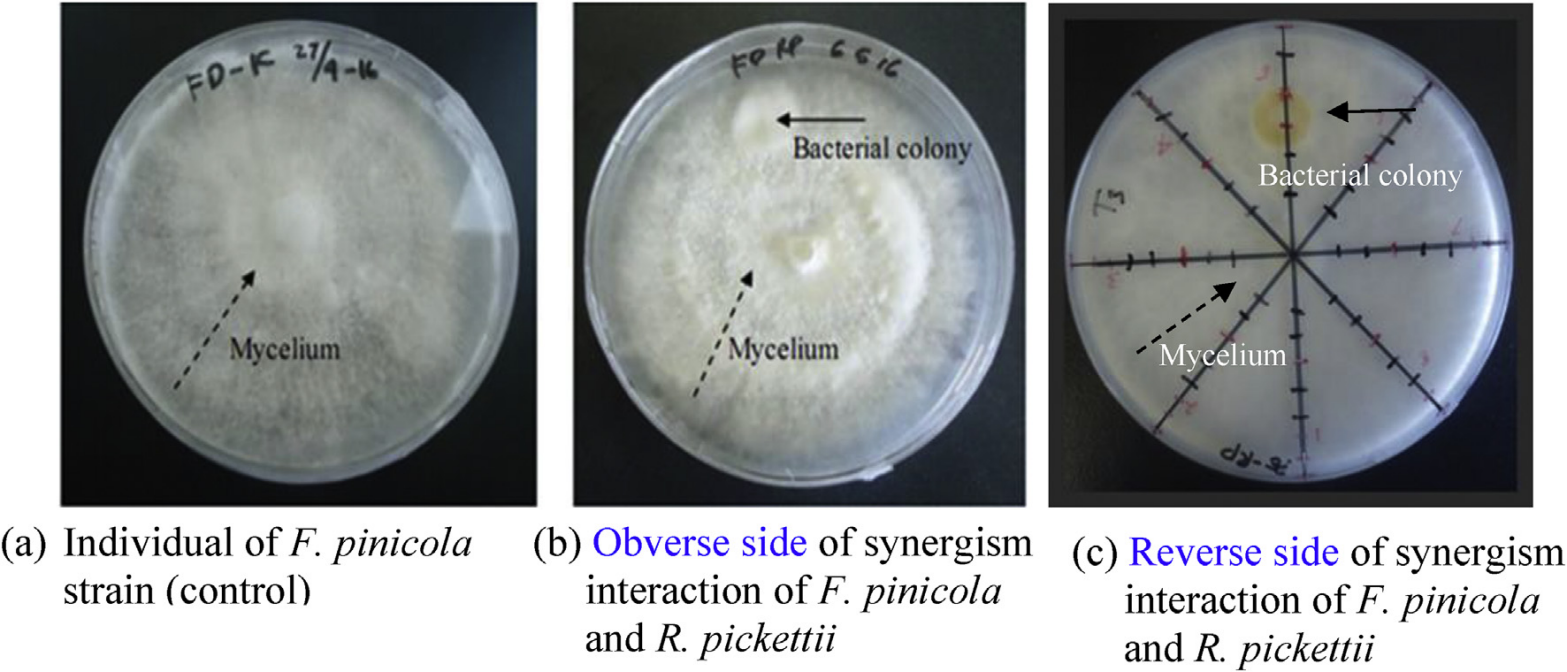
The mycelial growth of *F. pinicola* on PDA plates with or without inoculated bacterial cells.

In addition, fungal hyphae may function as transport vectors for bacterial (Ellegard-Jansen et al., 2014), which fungus has the ability to extend and penetrate through the distribution of hyphae (Trishul and Double, 2010; Jayekumar et al., 2013). *F. pinicola* and *R. pickettii* allow reciprocal growth from the onset, as fungal effusion in some cases, promotes substantial carbon supply for bacterial growth on the fungal hyphae (Ellegard-Jansen et al., 2014). The mycelial growth of *F. pinicola* on PDA with or without bacterial cell, by means of a confrontational assay, was distinguished and the results well documented (shown in Figure 4). When *F. pinicola* was incubated for 7 days with the bacterial strain *R. pickettii*, the growth of *F. pinicola* (Figure 4b) advanced in contrast to the its growth without bacterial cell (Figure 4a). When the mycelium of *F. pinicola* was growth with the bacterial colony, the bulky layer of hyphae was noticed nearby the bacterial cells. The presence of this bulky layer of hyphae perhaps accelerated the growth of aerial hyphae (Kamei et al., 2012). Furthermore, the growth rate of the mycelium of *F. pinicola* in the nearby bacterial cells was equivalent to the control prior to contact with the bacterial cells, but after contact with the mycelium of *F. pinicola*, there was a significant increase (data not shown). This may suggest that *R. pickettii* stimulated the mycelial growth of of *F. pinicola*, analogously as mycorrhizosphere bacteria with ectomycorrhizal fungi (Kamei et al., 2012).

The variation in the time of addition of the bacterium *R. pickettii*, as presented in Figure 2, shows that the addition of bacteria in the fungal culture on 0^th^ day results in the highest degradation of DDT. This indicates that the growth of bacteria and fungi together improves their respective biomass production through fungal-bacterial interactions (Bengstsson, 1992). Fungal and bacterial growth together in a microhabitat results in fungal hyphae providing additional exterior field area available for bacterial colonization (Romani et al., 2006; Gulis and Suberkropp, 2003). This result proves that *R. pickettii* grows better along with *F. pinicola* than individually, as the bacteria utilize the carbon substrates generated by the fungi (Romani et al., 2006). Notably, the addition of bacteria subsequently on days 1, 3 and 5 of incubation into *F. pinicola* culture also reduced DDT degradation, suggesting that *R. pickettii* acts on converted metabolic products of DDT. However, with abundant fungi in the stationary phase under longer incubation, the incubated fungi produced some secondary metabolites such as pinicolic acid and trametenolic acid to inhibit bacterial growth (Keller et al., 1996; Rosecke and Konig, 1999; Sariwati et al., 2017).

Metabolic products from DDT degradation by the consortium of *F. pinicola* and *R. pickettii* were analyzed by HPLC, on the basis of highest absorption at specific wavelength, compared to the standard retention time. DDE and DDD were identified as metabolic products of the biodegradation of DDT by *F. pinicola* (data not shown). DDE is effortlessly converted to DDD due to the presence of chlorine moieties and double bond which are highly electronegative. Since *R. pickettii* strain likewise generates DDE as its metabolic product, it was assumed that DDE was generated by both bacteria and fungi, which was later truncated to DDD by *F. pinicola*. DDMU was identified as another metabolic product (conducted by GCMS) aside from DDE and DDD, which was formed from DDE dechlorination and DDD dehydrochlorination reactions (Quensen et al., 1998). The consortia also utilized the capabilities of their extracellular enzymes to act together in the transformation of DDT, in which fungi metabolizes the initial step of oxidation, liberating to intermediate decomposition products that could be used by bacteria (Jacques et al., 2008). As the concentration of DDMU and DDE was lower than DDD (Table 2), DDD was assumed as the primary product of DDT biotransformation by the consortia.

To investigate the DDT degradation route, the metabolic products (DDD, DDE, and DDMU) of DDT degradation were used as the substrates (Table 2). The results showed that the degradation of DDD was lower than that of DDE, indicating that DDD was more resistant for degradation than DDE. DDD was degraded roughly by 50% and converted to DDE and DDMU, with the identified concentration of these metabolites less than 1%, suggesting there were undetected metabolites. As the concentration of DDE was higher than DDMU, DDD was converted to DDE and then to DDMU. On the other hand, DDE was degraded by roughly 55%, and converted to DDD (1%) and DDMU (5%), indicating that DDMU was the primary metabolite from DDE.

**Figure 5:**
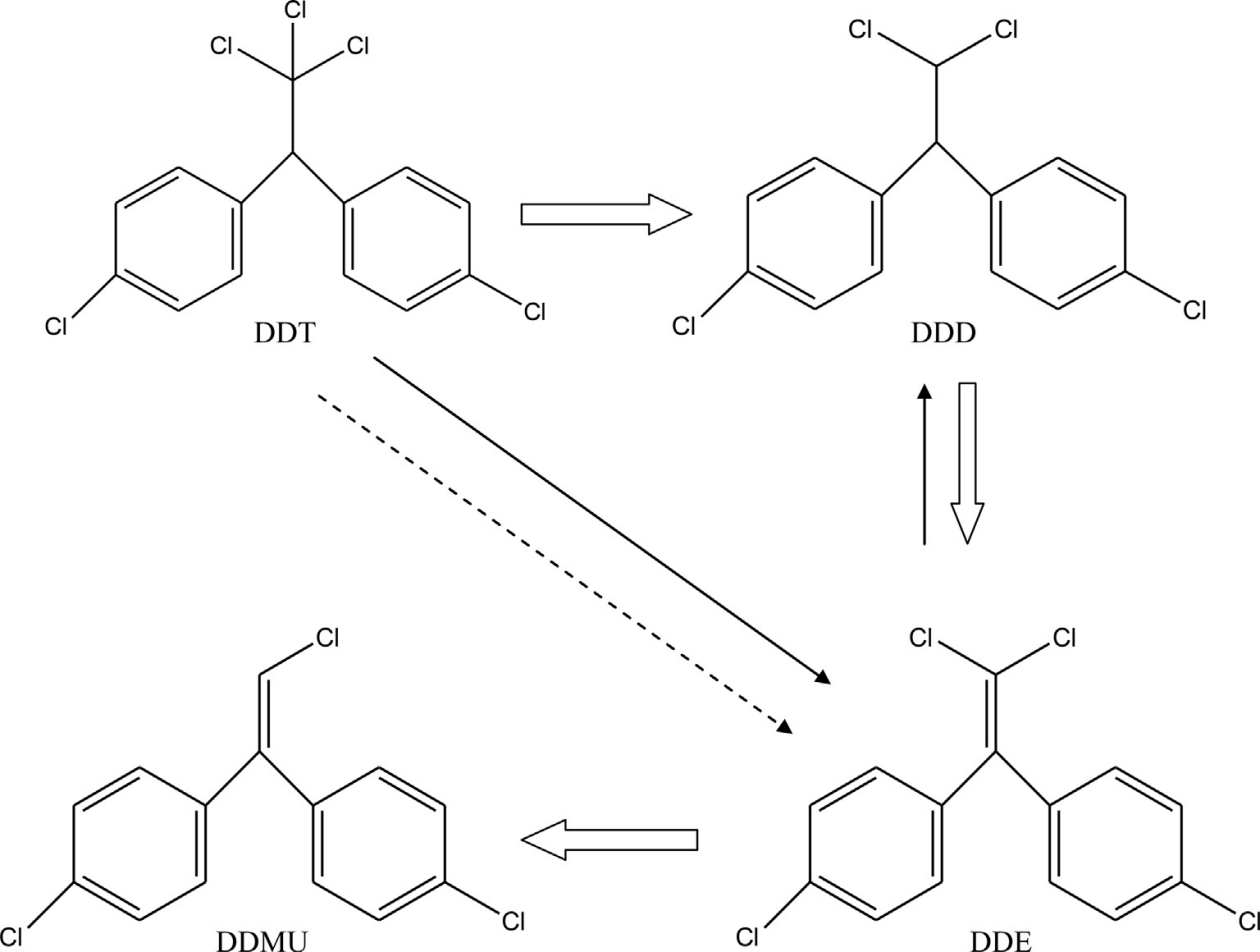
Proposed DDT degradation pathways by the brown-rot fungus *F. pinicola* only (thin black arrows), *R. pickettii* bacterium only (dotted arrows), and their co-cultures (open arrows).

Based on identification of metabolic products, the DDT degradation pathway by particular *F. pinicola* and *R. pickettii* as well as co-cultures was proposed (Figure 5). DDT was converted to DDE and DDD by *F. pinicola*, whilst DDT was converted to DDE by *R. pickettii*. Furthermore, *F. pinicola* transformed DDT to DDE, and then reduced DDE to DDD as the end product (Purnomo et al., 2008, 2010c, 2011a). DDT transformation through the synergistic degradation process resulted in modifications of the degradation pathway. DDT was transformed to DDD as its main metabolite via reductive dechlorination through single electron transfer, elimination of chlorine ion, and transformation of alkyl radical (Baxter, 1990; Foght et al., 2001) through the effects of *F. pinicola*. The fungal transformation of DDT to DDD as an end product (Purnomo et al., 2008) resulted in the high accumulation of DDD in the culture. Since *F. pinicola* could not transform DDD, further DDD degradation was carried out by *R. pickettii*, as DDD was transformed to DDE via dehydrogenation, followed by reductive de-chlorination of DDE to DDMU. Even, all of the identified metabolite compounds (DDD, DDE, DDMU) still had complex structures, which are also potential hazards to the environment. However, based on the LD_50_, the metabolites (DDD: 3,400.0 mg/kg; DDE: 880.9 mg/kg; DDMU: 2,700.0 mg/kg) are less toxic than DDT (113.0 mg/kg) (An et al., 2006; Hodgson et al., 2015).

## 5 Conclusion

The synergistic effect of *F. pinicola and R. pickettii* on the degradation of DDT was observed in this study. Degradation by *F. pinicola* in the absence of *R. pickettii* was relatively low (42%). The addition of 3 ml (1.44 × 10^13^ CFU/ml) of *R. pickettii* enhanced the effectiveness, degrading DDT by roughly 61%. Furthermore, DDE, DDD, and DDMU were metabolic products of DDT degradation by the synergistic system. Lastly, the DDT degradation route followed DDT conversion to DDD via reductive de-chlorination, further transformation of DDD to DDE by dehydrogenation, and then ultimate transformation of DDE into DDMU by de-chlorination.

## Declarations

### Author contribution statement

Adi Setyo Purnomo: Conceived and designed the experiments; Analyzed and interpreted the data; Contributed reagents, materials, analysis tools or data; Wrote the paper.

Atmira Sariwati: Performed the experiments; Analyzed and interpreted the data; Wrote the paper.

Ichiro Kamei: Analyzed and interpreted the data; Contributed reagents, materials, analysis tools or data; Wrote the paper.

### Funding statement

This work was supported by a grant from the research project for “INSINAS RISET PRATAMA INDIVIDU 2018, Number: 05/INS-1/PPKE4/2018” from the 10.13039/501100009509Ministry of Research, Technology and Higher Education, Indonesia under World Class University (WCU) Program managed by Institut Teknologi Bandung.

### Competing interest statement

The authors declare no conflict of interest.

### Additional information

No additional information is available for this paper.

## Appendix A Supplementary data

supplement revised.docx
