# Supplementary material for: Synergistic interaction of a consortium of the brown-rot fungus Fomitopsis pinicola and the bacterium Ralstonia pickettii for DDT biodegradation
Source: Heliyon. 2020 Jun 7;6(6):e04027. doi: 10.1016/j.heliyon.2020.e04027 (PMC7284076; doi:10.1016/j.heliyon.2020.e04027)

Supplementary 1

Pair test bacterium degradation and mixed cultures degradation


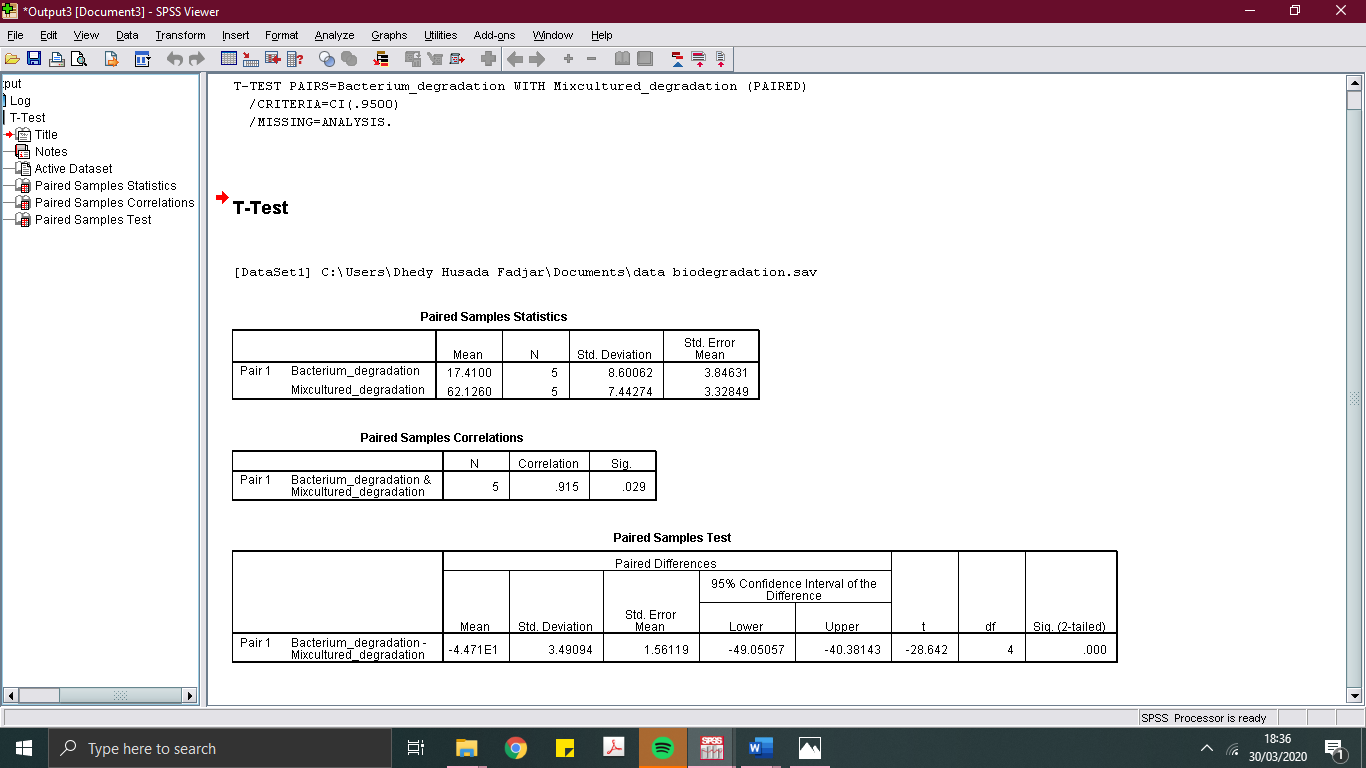


Supplementary 2

Pair test variation time


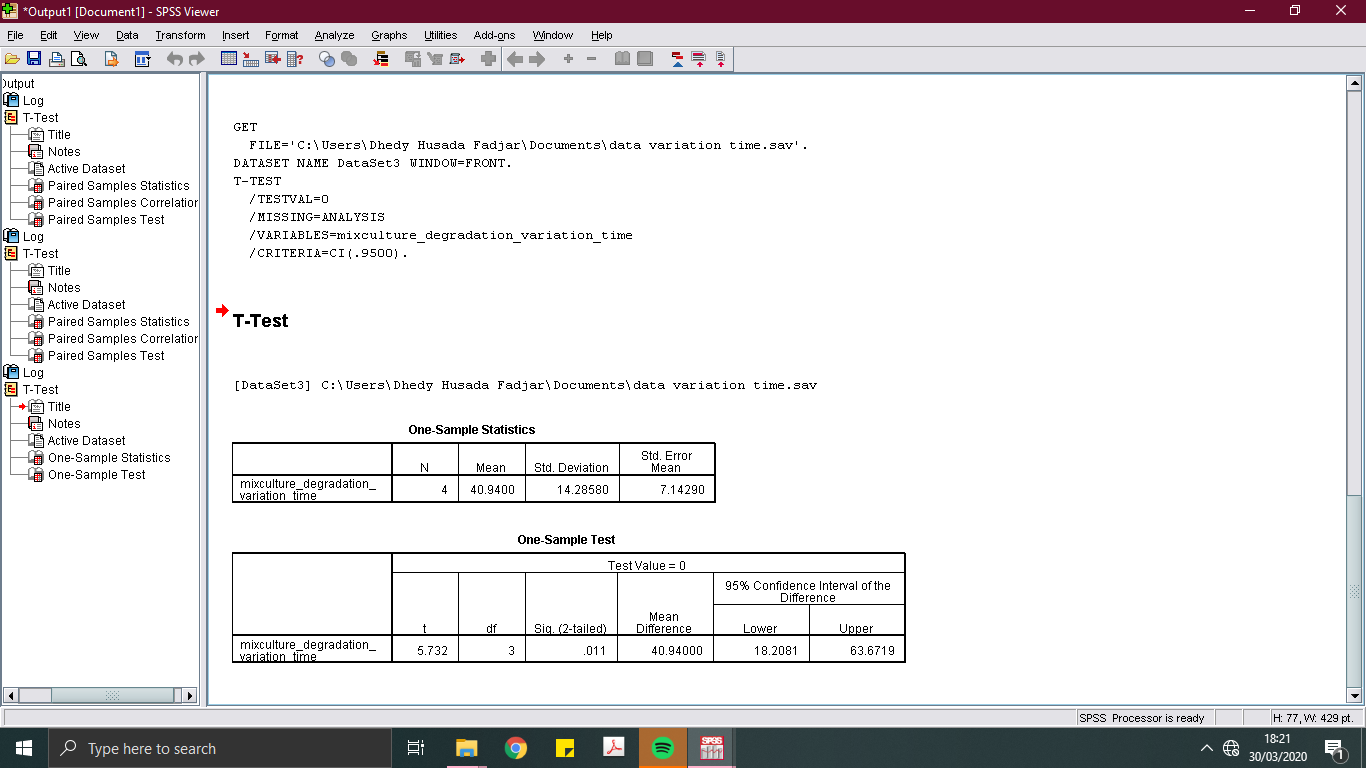

Supplement: Supplementary file 1 — supplement revised.docx [file mmc1.docx]
